# A trauma-informed care model for HIV prevention and care for refugee women in the United States: perspectives and implementation framework

**DOI:** 10.3389/fmed.2025.1537930

**Published:** 2025-03-19

**Authors:** Emmanuel Nazaire Essam Nkodo, Nada Fadul

**Affiliations:** Division of Infectious Diseases, Department of Internal Medicine, University of Nebraska Medical Center, Omaha, NE, United States

**Keywords:** HIV prevention and care, women, refugee, trauma informed care, implementation framework

## Abstract

HIV disproportionately impacts women, particularly in sub-Saharan Africa and other low-and middle-income countries, where conflict and displacement heighten their vulnerability to HIV. Refugee women face compounded challenges, including trauma before and during migration and healthcare inequities in host countries. This perspective paper aims to assess existing evidence on HIV treatment and prevention for refugee women resettling in the U.S., using intersectional stigma and the status-neutral service delivery model as theoretical frameworks, to propose an implementation strategy. Trauma-informed care (TIC) offers a promising approach to address these challenges, emphasizing culturally responsive, safe, and empowering healthcare. Integrating TIC with the status-neutral model, which centers on HIV testing and pathways for prevention or treatment, can improve care access and outcomes. Moreover, including refugee women and stakeholders in designing tailored interventions that address individual and systemic-level barriers is critical to fostering health equity.

## Introduction

HIV remains a significant global public health challenge, with approximately 39.9 million people living with HIV (PWH) by the end of 2023, 53% of whom are women and girls (1). Sub-Saharan Africa bears the highest burden, accounting for two-thirds of PWH globally, with women and girls representing 63% of new HIV infections (2). Globalization and increasing cross-border movements have reshaped HIV distribution patterns, often reflecting migration routes (3). With over 117 million forcibly displaced people globally as of 2023, Refugees—Persons fleeing to a place of safety to escape danger, persecution, or economic distress in their own country—represent a population at heightened risk for HIV infection (4). Forced migration, driven by factors such as oppression, economic hardship, sociopolitical marginalization, and armed conflict, exposes individuals – particularly refugee women—to severe trauma and heightened vulnerability to HIV (5, 6). This risk is compounded by conditions in countries of origin with high HIV prevalence, limited healthcare access during migration, and increased exposure to violence (7–9). Gender inequities, socioeconomic disadvantages, and the breakdown of protective social networks further exacerbate their susceptibility, leading to sexual and gender-based violence and exploitation such as transactional sex, which significantly amplifies their HIV risk (8). In 2023, the U.S. admitted 60,060 refugees, with 41% from Africa, 34% from Near East/South Asia, and 11% from Latin America/Caribbean; and 41.6% were women. This paper examines HIV prevention and care for refugee women in the U.S. and proposes a framework to improve services (4).

### The need for specialized HIV programs for refugee women

Host countries often lack the capacity or commitment to deliver adequate, culturally-sensitive care to refugee women, who must navigate unfamiliar healthcare systems during resettlement with limited support (5, 7). Many arrive with histories of trauma, including violence and displacement, which profoundly impact their physical and mental health (5). The United States, historically a leading refugee resettlement destination, has limited and fragmented research on the intersection of HIV and refugee women’s health, leaving critical gaps in understanding. Addressing these gaps requires tailored trauma-informed care (TIC) programs that integrate refugees’ unique vulnerabilities and social determinants of health, particularly in HIV prevention and care (5, 8). Such programs must address language barriers, limited educational opportunities in refugee camps, and the challenges of securing employment and housing in host countries. Culturally sensitive care respects and incorporates patients’ cultural beliefs, values, and experiences, ensuring services are adapted to diverse populations (10). Cultural considerations are often inconsistently applied and measured, as a 2011–2021 review found only 12% of HIV interventions were culturally sensitive (11) Understanding these overlapping challenges is essential to dismantling the systemic and structural obstacles to HIV equity for this population.

### Intersectional stigma: a theoretical lens

Intersectional stigma provides a powerful framework for analyzing and addressing the barriers encountered by refugee women. This theory highlights that stigmatization does not occur in isolation but rather as a complex web of intersecting societal oppressions (12). For example, a refugee woman living with HIV may simultaneously face HIV-related stigma, characterized by fear of ostracism within her community; gender stigma, which limits her autonomy in healthcare decision-making; racial stigma, manifesting as discrimination within healthcare systems; and migrant stigma, driven by fears of being perceived as a burden or outsider in the host country, as some are often accused of importing HIV in the host countries (13). The literature shows that African immigrants and refugees have HIV infection rates six times higher than any other minority group in the U.S. (14). Migrant stigma related to HIV is particularly pronounced in refugee populations, as they are often scapegoated for bringing the virus into host communities (3, 9). This discriminatory perception can further leads refugees to neglecting their health needs, while survival priorities—such as securing food, safety, and shelter—frequently take precedence over seeking healthcare, delaying HIV testing and treatment (3, 5, 15). Moreover, language barriers and cultural stigma further exacerbate these issues, leading to delayed care initiation and poor treatment retention (5, 7, 16). These overlapping stigmas compound to erode trust in healthcare providers, delay testing, and reduce engagement in care (15–17). Furthermore, historical exclusionary policies related to HIV have deepened these fears, leaving a legacy of mistrust that persists even after policy changes (3). Additionally, refugees often reside in crowded living conditions where inadvertent disclosure of HIV status can lead to social exclusion or discrimination (18). This fear is particularly acute for women, who may experience compounded stigma. These cultural dynamics intersect with additional structural and systemic barriers, making healthcare access even more challenging. Intersectional stigma theory helps to explain how these overlapping stigmas magnify the challenges of accessing HIV testing and care as women navigate a healthcare system that often lacks culturally sensitive approaches.

### A status-neutral HIV prevention and care continuum for refugee women

The status-neutral model offers a novel framework for the HIV continuum of care integrating both PWH and those at risk of HIV (19). Beginning with an HIV test, the model divides into two pathways: “HIV Primary Prevention Engagement” for those testing negative and “HIV Treatment Engagement” for those testing positive. Both pathways share a common goal: sustained engagement in clinical care to minimize HIV transmission or acquisition risk. The model emphasizes that preventive and quality care services require continuous collaboration between patients and providers to sustain engagement in care or treatment.

**Figure 1:**
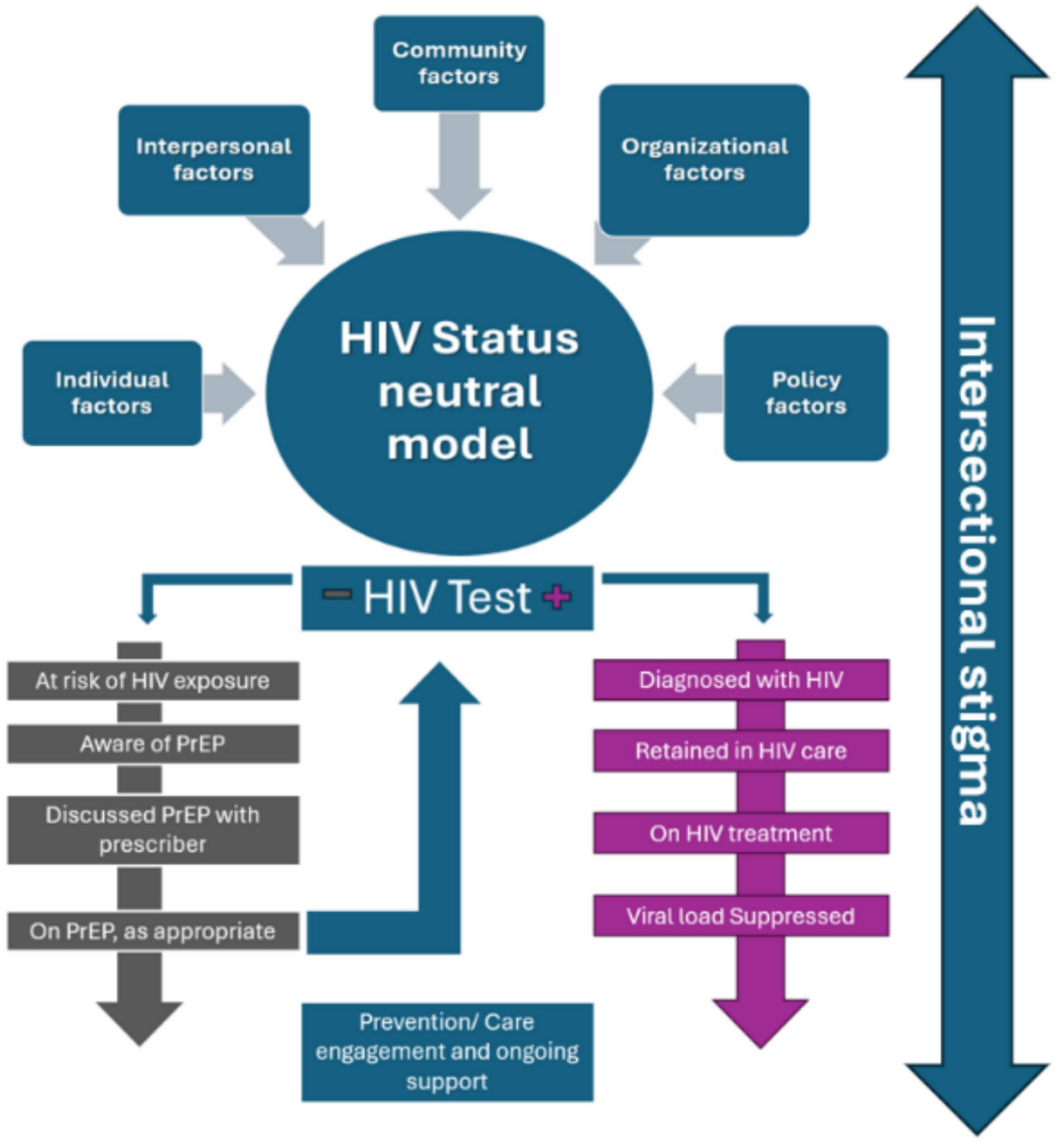
Status-neutral HIV care and prevention model. This figure illustrates the interplay of various factors that impact refugee women throughout the continuum.

Applying the status-neutral model to refugee women, requires addressing the intricate interplay of factors across multiple levels (Figure 1):

- Individual level: studies have shown inconsistencies between knowledge of HIV acquisition and behaviors relating to HIV positive individuals among US based refugee women from Africa, which may indicate limited understanding of HIV transmission and prevention often shaped by cultural beliefs or lack of education (20, 21)
- Interpersonal level: norms from the country of origin around HIV prevention and care, gender-based power imbalances, fear of partner violence, and stigma within families can hinder testing and treatment engagement (21).
- Community Level: Discrimination against refugees and individuals living with HIV, as well as cultural taboos surrounding discussions of sexual health limit awareness and access to services (8, 19, 21).
- Organizational level: language barriers remain a major challenge. While Spanish translation is common in U.S. healthcare, many refugee languages, like Burmese and African dialects, are underrepresented (16). Additionally, providers often lack trauma-informed training, as TIC is still emerging in refugee healthcare, limiting awareness and capacity (22). Social support, culturally safe providers, and patient navigators can improve comfort, knowledge, participation, and trust in host country health system (5, 17, 23).
- Policy level: refugee women face healthcare access barriers due to complex eligibility tied to immigration status. While the Refugee Medical Assistance (RMA) program offers short-term coverage, gaps emerge if they do not qualify for Medicaid or state programs. The Ryan White HIV/AIDS Program covers PWH regardless of status but is limited to HIV care, excluding preventive services like PrEP. Expanding access to public and private insurance is crucial for improving preventive healthcare.

Acknowledging and addressing these interconnected factors is critical to improving outcomes and ensuring the success of this initiative (Figure 1).

Historically, public health responses to the needs of refugee women have been scarce. Existing TIC efforts in the US primarily focused on addressing issues such as intimate partner violence (IPV), domestic abuse, and child abuse, with a limited number of interventions or research reflecting HIV and TIC among refugee women highlighting the need for tailored interventions for that vulnerable group (17, 24). Moreover, existing, efforts are often hampered by logistical challenges, under-resourced health systems, and a lack of targeted interventions that consider the specific needs of displaced women (3, 7).

### Structural and cultural barriers to HIV testing

HIV testing is a critical entry point for the implementation of the status-neutral HIV service delivery model. The Center for Diseases Prevention and Control (CDC) recommends routine opt-out HIV screening for refugees and immigrants as part of post-arrival healthcare (3). However, data on adherence to these guidelines and implementation outcomes remain limited. For testing to occur, women must be aware of their HIV risk, have access to primary care, sexually transmitted infection (STI) clinics, or other healthcare settings, and consult with culturally sensitive health providers. Studies looking at refugee women’s Knowledge, Attitudes, and Practices (KAP) reveal that many women have low knowledge and perception of their own HIV risk due to cultural or contextual factors, further reducing testing rates (19, 21, 25). Structural barriers such as lack of insurance, transportation challenges, and fragmented healthcare systems significantly limit access to care and contribute to the low rates of HIV testing and counseling among refugee and immigrant women (23). Additionally, intersectional stigma—stemming from the interplay of race, ethnicity, gender, HIV status, forced displacement, and refugee identity—creates fear and hesitancy around seeking testing (mistrust in how asylum host countries may use this information and difficulty in maintaining confidentiality in refugee settings) (3, 18).

Prenatal care presents another critical opportunity, as HIV testing is mandated during pregnancy. However, studies have identified missed opportunities, as refugee status has been associated with the late presentation of HIV (26). While women testing positive during prenatal care can be linked to care with good outcomes, those testing negative often receive minimal counseling on HIV prevention. Language barriers exacerbate these issues, as limited English proficiency hinders effective communication with healthcare providers. Compounding these challenges is the frequent lack of trauma-informed and culturally responsive care, as many healthcare providers are not trained to address the unique needs and lived experiences of refugee populations. Strategies that increase the ease of accessing HIV testing will likely result in decreased morbidity, mortality, and transmission of disease among this population. Paramount to that, gaps in KAP should be addressed, as studies showed inadequate knowledge of HIV transmission and inconsistencies between knowledge of HIV acquisition and behaviors relating to HIV-positive individuals, which may indicate HIV-associated stigma among refugee women (19, 25).

Despite these challenges, several strategies have shown promise in improving HIV testing and reducing the stigma around it. Studies on social support and its impact on HIV testing and adherence among refugee populations showed that informational support encouraged refugees to test for HIV (27).

### Engagement in HIV prevention

Prevention strategies for HIV among refugee women must be culturally sensitive and context-specific, recognizing the unique challenges faced by this population. A review of various HIV prevention interventions highlights the importance of integrating cultural beliefs and experiences into program designs to improve their effectiveness (28). This is particularly crucial for refugee women who may have different social norms and barriers to health care access. Educational interventions hold great promise as a study by Cianelli et al. showed that increased HIV knowledge positively correlates with self-efficacy in HIV prevention, meaning women with more knowledge are more confident in their ability to adopt HIV prevention measures (29).

Culturally tailored programs that engage community members as peer leaders have proven effective in promoting HIV prevention strategies. Such participatory approaches not only enhance knowledge but also build social capital, which is critical for facilitating access to health services among refugee populations (9, 11, 16). Programs must provide information about available health services within refugee settlements, ensuring that women are aware of their options for prevention and treatment (5).

Furthermore, it is essential to incorporate strong social and psychological support systems from families, communities, and healthcare providers to improve adherence to HIV care within these settings (5). Promoting awareness and education among men and boys regarding sexual and reproductive health and gender equity is another critical component of HIV prevention strategies (3). By fostering gender equity, interventions can reduce stigma and discrimination, thereby facilitating more effective HIV prevention efforts for refugee women.

### Engagement in HIV treatment

Research on the HIV continuum of care for refugee women in the U.S. remains limited, but studies from other countries provide valuable insights into the challenges these women face in engaging with HIV care. Pregnancy often serves as a pivotal entry point and represent the first sustained interaction with formal healthcare services, as it necessitates medical attention for maternal and child health needs (26, 30, 31). However, recent studies suggest that the prevalence of HIV among refugee women is typically higher than that of pregnant women in host countries, underscoring the urgency of targeted interventions (30). Furthermore, research indicated that refugee women who access antenatal visits face an increased risk of delayed HIV diagnosis due to their migrant background, which can jeopardize the health of both the mother and the unborn baby (26). High HIV prevalence in this population is further associated with severe maternal morbidity, emphasizing the importance of early and consistent HIV testing as a life-saving measure (31).

Studies exploring ART use among pregnant refugee women with HIV highlight significant factors influencing non-adherence. Late initiation of prevention of mother to child transmission (PMTCT) services in the third trimester reduces the time available for viral suppression, psychological adjustment, and treatment adherence. Additionally, women not requiring partner or family permission for PMTCT services exhibited lower adherence, likely reflecting insufficient social support (13). Negative attitudes from healthcare providers further diminished adherence, emphasizing the need for better training and support for service providers to foster trust and engagement.

The intersection of pregnancy, HIV and the cultural and systemic challenges of resettlement compounds the vulnerability of refugee women, who often conceal their HIV diagnosis due to stigma (32). Social support plays a crucial role in mitigating these challenges. For example, a study by Rouhani et al. in Southwestern Uganda demonstrated the dual importance of emotional support, which helps individuals cope with HIV diagnoses, and instrumental support, which facilitates ART adherence (27). Refugee women living with HIV also acted as agents of change by educating their social networks promoting testing, and accompanying others to testing sites. These findings underscore the importance of involving refugee women living with HIV in the design and implementation of programs to improve testing and treatment access for their social network.

### Implementation of trauma-informed HIV care model for refugee women

Trauma-informed care for refugee women addresses the profound effects of trauma stemming from experiences during displacement, conflict, or resettlement. Refugee women face unique challenges, including high rates of sexual and intimate partner violence during migration, leading to mental health issues such as post-traumatic stress disorder (PTSD) (5, 33, 34). Women with lifetime trauma are more likely to report PTSD, depression, anxiety, and substance use, which negatively impact their quality of life and adherence to antiretrovirals (35). This care model emphasizes creating a safe, trusting healthcare environment that focuses on cultural sensitivity and patient empowerment (36). Such models should aim to avoid re-traumatization and facilitate healing through comprehensive, interdisciplinary approaches. TIC is particularly relevant for HIV care, as trauma is prevalent among people with or at risk for HIV and contributes to HIV acquisition, morbidity, and mortality. NASTAD’s Trauma-Informed Approaches Toolkit is a valuable resource that offers guidelines for healthcare providers on implementing TIC within HIV care systems. The toolkit emphasizes the importance of healing-centered care, recognizing that achieving viral suppression is just one aspect of comprehensive HIV treatment (36).

Few studies have examined the impact of TIC interventions on HIV care outcomes among this population. However, recent findings suggest that a trauma-informed approach to developing interventions may help to improve treatment outcomes, such as engagement in care and adherence to antiretrovirals (37). Research shows that social support is essential in reducing feelings of isolation and improving healthcare access for refugee women (5). Interventions like the “Tree of Life” for women with HIV have shown potential in helping refugee women identify personal strengths and qualities that enabled them to cope and build their resilience by empowering them to re-author their life narratives (38).

Despite its significance, the implementation of TIC faces considerable challenges, including systemic barriers such as limited access to mental health services, cultural stigma surrounding mental health, and language barriers that can complicate communication between providers and refugee women (33, 39, 40). Additionally, inadequate training among healthcare professionals in trauma-specific practices can hinder effective care delivery, underscoring the necessity for ongoing education and policy advocacy to support this population (22, 41).

We propose applying the Consolidated Framework for Implementation Research (CFIR) to guide the systematic implementation of TIC for refugee women living with or at risk of HIV (42). The CFIR framework allows for a structured approach to identifying and addressing individual, organizational, and policy-level determinants influencing the implementation process (Table 1). The CFIR was chosen for its multi-level approach, considering individual, organizational, and policy factors in implementing TIC (43). Unlike frameworks focused solely on behavior change or readiness, CFIR systematically assesses barriers and facilitators, making it ideal for complex interventions like trauma-informed, status-neutral HIV care for refugee women. However, its main limitation is the lack of guidance on adapting interventions based on identified factors.

**Table 1:** Using the consolidated framework for implementation research (CFIR) to guide the systematic implementation of trauma-informed care for refugee women living with or at risk of HIV.

Implementation of trauma informed care in HIV prevention and care for US refugee women				
Characteristics of the intervention	Outer setting	Inner setting	Individuals involved	Implementation process
Highlight robust evidence showing the benefits of TIC in improving health outcomes for trauma-affected populations. Use studies demonstrating the link between trauma-informed approaches and increased adherence to HIV prevention and care. Customize TIC principles (safety, trustworthiness, peer support, collaboration, empowerment, and cultural relevance) to the needs of refugee women. Address unique cultural and linguistic needs and adapt interventions to specific refugee contexts. Acknowledge the multi-layered nature of TIC, requiring training for staff, adjustments to clinic environments, and system-level changes. Simplify initial implementation with clear guidelines and phased approaches. Secure funding for training, resources, and additional staff to ensure TIC is sustainable. Highlight cost-effectiveness by linking improved patient outcomes to reduced healthcare utilization costs.	Assess the trauma experiences, mental health challenges, and HIV-related stigma refugee women face. Provide culturally sensitive education on HIV prevention and trauma-informed services. Leverage support from public health policies and grants targeting vulnerable populations. Advocate for inclusive healthcare policies that address the needs of refugees. Benchmark with other organizations successfully using TIC in similar settings. Collaborate with refugee advocacy groups and local public health entities to promote acceptance of TIC.	Build a culture prioritizing TIC by promoting its alignment with organizational goals of improving HIV care for underserved populations. Create incentives for staff participation and engagement in TIC-related initiatives. Provide training to enhance staff knowledge and skills in trauma-informed practices. Ensure resources such as interpreters, private counseling spaces, and culturally sensitive materials are available. Strengthen communication between leadership, frontline workers, and external stakeholders to support consistent TIC practices. Use feedback loops to identify and address barriers in real time.	Address misconceptions among staff about TIC and its relevance to HIV prevention and care. Use workshops and success stories to build buy-in and confidence in TIC. Provide ongoing training and support to build staff confidence in delivering trauma-informed care. Encourage staff to view TIC as a key part of their role in providing equitable and compassionate care.	Conduct a needs assessment to understand the specific challenges and opportunities in integrating TIC for refugee women. Develop an actionable plan with defined goals, timelines, and evaluation metrics. Involve refugee women as co-designers of services to ensure cultural and contextual appropriateness. Engage stakeholders like community organizations, policy advocates, and healthcare providers. Pilot TIC in a few clinics or programs before scaling up. Ensure fidelity to TIC principles while remaining flexible for contextual adaptations. Use qualitative and quantitative measures to assess the impact of TIC on patient outcomes, staff satisfaction, and system-level changes. Continuously refine the implementation strategy based on feedback and results.

The co-design of a tailored action plan, informed by the needs assessment, should actively engage refugee women and key stakeholders to ensure cultural relevance and stakeholder buy-in. Developing implementation outcomes, such as engagement in prevention and care programs, is crucial for evaluating success. Continuous quality improvement can enhance fidelity and sustainability by enabling ongoing adaptation to systemic, organizational, and policy environments. This evidence-based approach ensures that TIC implementation is robust, scalable, and effective in meeting the needs of refugee women.

## Conclusion

Efforts to address HIV prevention and care for refugee women must account for their unique challenges, including heightened vulnerability due to migration-related trauma, limited healthcare access, and stigmas. Trauma-informed care (TIC) plays a pivotal role in addressing both immediate mental health needs and the broader emotional well-being of refugee women and their families. Combining the HIV status-neutral service delivery model with TIC can significantly enhance access to testing, prevention, and treatment services, offering culturally sensitive, tailored interventions. To implement such approaches effectively, the CFIR framework can guide efforts to address systemic and individual-level barriers essential for ensuring equitable healthcare access and improving outcomes for refugee women, bringing us closer to ending the HIV epidemic.

## Data Availability

The original contributions presented in the study are included in the article/supplementary material, further inquiries can be directed to the corresponding author.

## References

[1] UNAIDS. Global HIV statistics. (2024). Available at: https://www.unaids.org/sites/default/files/media_asset/UNAIDS_FactSheet_en.pdf (Accessed November 22, 2024)

[2] Thematic_fs_hiv_girls_women.pdf. (n.d.). Available at: https://thepath.unaids.org/wp-content/themes/unaids2023/assets/files/thematic_fs_hiv_girls_women.pdf (Accessed November 22, 2024)

[3] CDC. (2024). HIV infection. Immigrant and Refugee Health. Available online at: https://www.cdc.gov/immigrant-refugee-health/hcp/domestic-guidance/hiv-infection.html

[4] Schofield Noah Yap Amanda. 2024_1108_ohss_refugee_annual_flow_report_2023.pdf. (n.d.). Retrieved February 14, 2025. Available online at: https://ohss.dhs.gov/sites/default/files/2024-11/2024_1108_ohss_refugee_annual_flow_report_2023.pdf

[5] Hawkins MM Schmitt ME Adebayo CT Weitzel J Olukotun O Christensen AM. Promoting the health of refugee women: a scoping literature review incorporating the social ecological model. Int J Equity Health. (2021) 20:45. doi: 10.1186/s12939-021-01387-5, PMID: 33485342 PMC7825239

[6] Palattiyil G Sidhva D Seraphia Derr A Macgowan M. Global trends in forced migration: policy, practice and research imperatives for social work. Int Soc Work. (2022) 65:1111–29. doi: 10.1177/00208728211022791

[7] Davidson N Hammarberg K Romero L Fisher J. Access to preventive sexual and reproductive health care for women from refugee-like backgrounds: a systematic review. BMC Public Health. (2022) 22:403. doi: 10.1186/s12889-022-12576-4, PMID: 35220955 PMC8882295

[8] Stirling-Cameron E Almukhaini S Dol J DuPlessis BJ Stone K Aston M. Access and use of sexual and reproductive health services among asylum-seeking and refugee women in high-income countries: a scoping review. PLoS One. (2024) 19:e0312746. doi: 10.1371/journal.pone.0312746, PMID: 39509374 PMC11542864

[9] Zhang X Rhoads N Rangel MG Hovell MF Magis-Rodriguez C Sipan CL. Understanding the impact of migration on HIV risk: an analysis of Mexican migrants’ sexual practices, partners, and contexts by migration phase. AIDS Behav. (2017) 21:935–48. doi: 10.1007/s10461-016-1622-4, PMID: 27888370 PMC5837820

[10] Claeys A Berdai-Chaouni S Tricas-Sauras S De Donder L. Culturally sensitive care: definitions, perceptions, and practices of health care professionals. J. Trans. Nurs. (2021) 32:484–92. doi: 10.1177/1043659620970625, PMID: 33150857

[11] Vitsupakorn S Pierce N Ritchwood TD. Cultural interventions addressing disparities in the HIV prevention and treatment cascade among black/African Americans: a scoping review. BMC Public Health. (2023) 23:1748. doi: 10.1186/s12889-023-16658-9, PMID: 37679765 PMC10485990

[12] Thompson VE. Intersectionality (Key concepts 2nd edition). In: Ethnic and racial studies vol. 44. (2020) p. 1472–1474.

[13] Tusabe J Nangendo J Muhoozi M Muyinda H. Use and non-adherence to antiretroviral therapy among refugee HIV positive pregnant mothers aged 18–49 years in Kyangwali refugee camp, Western Uganda. AIDS Res Ther. (2024) 21:54. doi: 10.1186/s12981-024-00645-0, PMID: 39175044 PMC11340033

[14] Moore S Zajicek-Farber ML Donaldson LP. A snapshot of HIV/AIDS knowledge, behaviors, and attitudes of Ethiopian immigrants in the District of Columbia. J Ethn Cult Divers Soc Work. (2024) 33:110–20. doi: 10.1080/15313204.2022.2154879

[15] Darebo TD Spigt M Teklewold B Badacho AS Mayer N Teklewold M. The sexual and reproductive healthcare challenges when dealing with female migrants and refugees in low and middle-income countries (a qualitative evidence synthesis). BMC Public Health. (2024) 24:520. doi: 10.1186/s12889-024-17916-0, PMID: 38373954 PMC10877851

[16] Vu M Besera G Ta D Escoffery C Kandula NR Srivanjarean Y. System-level factors influencing refugee women’s access and utilization of sexual and reproductive health services: a qualitative study of providers’ perspectives. Front. Glob. Women Health. (2022) 3:1048700. doi: 10.3389/fgwh.2022.1048700, PMID: 36589147 PMC9794861

[17] Mathis CM Steiner JJ Kappas Mazzio A Bagwell-Gray M Wachter K Johnson-Agbakwu C. Sexual and reproductive healthcare needs of refugee women exposed to gender-based violence: the case for trauma-informed Care in Resettlement Contexts. Int J Environ Res Public Health. (2024) 21:1046. doi: 10.3390/ijerph21081046, PMID: 39200656 PMC11355007

[18] Correa-Salazar C Amon JJ Page K Groves AK Bilal U Vera A. Barriers and facilitators to HIV prevention and care for Venezuelan migrant/refugee women and girls in Colombia. Jo. Migr. Health. (2023) 8:100206. doi: 10.1016/j.jmh.2023.100206, PMID: 38047140 PMC10690627

[19] Myers JE Braunstein SL Xia Q Scanlin K Edelstein Z Harriman G. Redefining prevention and care: a status-neutral approach to HIV. Open Forum Infect Dis. (2018) 5:ofy097. doi: 10.1093/ofid/ofy097, PMID: 29977957 PMC6016418

[20] Agbemenu K Aidoo-Frimpong G Auerbach S Jafri A. HIV attitudes and beliefs in U.S.-based African refugee women. Ethn Health. (2022) 27:499–508. doi: 10.1080/13557858.2020.1740175, PMID: 32228028

[21] Feresu S Smith L. Knowledge, attitudes, and beliefs about HIV/AIDS of Sudanese and bantu Somali immigrant women living in Omaha, Nebraska. Open J Prev Med. (2013) 3:84–98. doi: 10.4236/ojpm.2013.31011

[22] Im H Swan LET. Working towards culturally responsive trauma-informed Care in the Refugee Resettlement Process: qualitative inquiry with refugee-serving professionals in the United States. Behav. Sci. (2021) 11:9. doi: 10.3390/bs11110155, PMID: 34821616 PMC8614655

[23] Li K Thaweesee N Kimmel A Dorward E Dam A. Barriers and facilitators to utilizing HIV prevention and treatment services among migrant youth globally: a scoping review. PLOS Glob. Public Health. (2024) 4:e0002851. doi: 10.1371/journal.pgph.0002851, PMID: 38354206 PMC10866458

[24] Sales JM Swartzendruber A Phillips AL. Trauma-informed HIV prevention and treatment. Curr HIV/AIDS Rep. (2016) 13:374–82. doi: 10.1007/s11904-016-0337-5, PMID: 27704251 PMC5107145

[25] Khan MN Rahman MM Rahman MM Islam MM. HIV transmission knowledge among Rohingya refugee women in Bangladesh: a cross-sectional survey. BMJ Open. (2021) 11:e047516. doi: 10.1136/bmjopen-2020-047516, PMID: 34598982 PMC8488728

[26] Singer K Schulze-Sturm U Alba-Alejandre I Hollwitz B Nguyen TTT Sollinger F. Impact of refugee influx on the epidemiology of late-presenting HIV-infected pregnant women and mother-to-child transmission: comparing a southern and northern medical Centre in Germany. Infection. (2019) 47:847–52. doi: 10.1007/s15010-019-01332-3, PMID: 31190299

[27] Rouhani SA O’Laughlin KN Faustin ZM Tsai AC Kasozi J Ware NC. The role of social support on HIV testing and treatment adherence: a qualitative study of HIV-infected refugees in southwestern Uganda. Glob Public Health. (2017) 12:1051–64. Scopus. Doi:10.1080/17441692.2015.1132472. doi: 10.1080/17441692.2015.1132472, PMID: 26783835 PMC4955653

[28] Wyatt GE Williams JK Gupta A Malebranche D. Are cultural values and beliefs included in U.S. based HIV interventions? Prev Med. (2012) 55:362–70. doi: 10.1016/j.ypmed.2011.08.021, PMID: 21884721 PMC3736836

[29] Cianelli R Villegas N McCabe BE de Tantillo L Peragallo N. Self-efficacy for HIV prevention among refugee Hispanic women in South Florida. J Immigr Minor Health. (2017) 19:905–12. doi: 10.1007/s10903-016-0462-7, PMID: 27470226 PMC5659853

[30] Goosen S Hoebe CJPA Waldhober Q Kunst AE. High HIV prevalence among asylum seekers who gave birth in the Netherlands: a Nationwide study based on antenatal HIV tests. PLoS One. (2015) 10:e0134724. doi: 10.1371/journal.pone.0134724, PMID: 26296093 PMC4546638

[31] Wanigaratne S Cole DC Bassil K Hyman I Moineddin R Urquia ML. Contribution of HIV to maternal morbidity among refugee women in Canada. Am J Public Health. (2015) 105:2449–56. doi: 10.2105/AJPH.2015.302886, PMID: 26469648 PMC4638260

[32] Chulach T Gagnon M Holmes D. The lived experience of pregnancy among HIV-positive refugee women: a qualitative study. ANS Adv Nurs Sci. (2016) 39:130–49. doi: 10.1097/ANS.0000000000000117, PMID: 27149227

[33] DeSa S Gebremeskel AT Omonaiye O Yaya S. Barriers and facilitators to access mental health services among refugee women in high-income countries: a systematic review. Syst Rev. (2022) 11:62. doi: 10.1186/s13643-022-01936-1, PMID: 35387680 PMC8985267

[34] Wylie L Van Meyel R Harder H Sukhera J Luc C Ganjavi H. Assessing trauma in a transcultural context: challenges in mental health care with immigrants and refugees. Public Health Rev. (2018) 39:22. doi: 10.1186/s40985-018-0102-y, PMID: 30151315 PMC6103972

[35] Cuca YP Shumway M Machtinger EL Davis K Khanna N Cocohoba J. The association of trauma with the physical, behavioral, and social health of women living with HIV: pathways to guide trauma-informed health care interventions. Women’s Health Issues: Official Publication of the Jacobs Institute of Women’s Health. (2019) 29:376–384. doi: 10.1016/j.whi.2019.06.001, PMID: 31303419 PMC6755036

[36] Using Trauma-Informed Approaches to End the HIV Epidemic | NASTAD. (2023). Available online at: https://nastad.org/blog/using-trauma-informed-approaches-end-hiv-epidemic

[37] Brown MJ Adeagbo O. Trauma-informed HIV care interventions: towards a holistic approach. Curr HIV/AIDS Rep. (2022) 19:177–83. doi: 10.1007/s11904-022-00603-3, PMID: 35353271 PMC10084732

[38] Vitale A Khawaja NG Ryde J. Exploring the effectiveness of the tree of life in promoting the therapeutic growth or refugee women living with HIV. Arts Psychother. (2019) 66:101602. doi: 10.1016/j.aip.2019.101602

[39] Bailey T. (2017). Enhancing evidence-based interventions for refugees. Trauma Psychology News. Available online at: https://traumapsychnews.com/2017/07/enhancing-evidence-based-interventions-for-refugees/

[40] Moezzi SMI Etemadi M Lankarani KB Behzadifar M Katebzada H Shahabi S. Barriers and facilitators to primary healthcare utilization among immigrants and refugees of low and middle-income countries: a scoping review. Glob Health. (2024) 20:75. doi: 10.1186/s12992-024-01079-z, PMID: 39449084 PMC11515291

[41] Policy Guide: Improving Access to Mental Healthcare for Refugees and Other Displaced People in the United States. (n.d.). The Refugee Advocacy Lab. Retrieved November 26, 2024. Available online at: https://www.refugeeadvocacylab.org/resources/mental-health-policy-guide

[42] Damschroder LJ Aron DC Keith RE Kirsh SR Alexander JA Lowery JC. Fostering implementation of health services research findings into practice: a consolidated framework for advancing implementation science. Implement Sci. (2009) 4:50. doi: 10.1186/1748-5908-4-50, PMID: 19664226 PMC2736161

[43] Piper KN Brown LL Tamler I Kalokhe AS Sales JM. Application of the consolidated framework for implementation research to facilitate delivery of trauma-informed HIV care. Ethn Dis. (2021) 31:109–18. doi: 10.18865/ed.31.1.109, PMID: 33519161 PMC7843045

